# Spatial and Temporal Disparity Analyses of Glycosylated Benzaldehyde and Identification and Expression Pattern Analyses of Uridine Diphosphate Glycosyltransferase Genes in *Prunus mume*

**DOI:** 10.3390/plants13050703

**Published:** 2024-03-01

**Authors:** Haotian Jia, Xiaoyun Geng, Lina Fan, Xin Li, Jiao Wang, Ruijie Hao

**Affiliations:** College of Horticulture, Shanxi Agricultural University, Jinzhong 030600, China; a1281279089@163.com (H.J.); gxy121721@163.com (X.G.); 16634253802@163.com (L.F.); 15536449736@163.com (X.L.); 18212075833@163.com (J.W.)

**Keywords:** benzaldehyde, GBVs (glycoside-bound volatiles), *Prunus mume*, UGTs (uridine diphosphate glycosyltransferase)

## Abstract

The species *Prunus mume* consists of uniquely aromatic woody perennials with large amounts of free aromatic substances in the flower cells. Uridine diphosphate glycosyltransferase (UGT) modifies these free aromatic substances into water-soluble glycoside-bound volatiles (GBVs) which play an important role in regulating the use of volatiles by plants for information exchange, defense, and stress tolerance. To investigate the changes in the glycosidic state of aromatic substances during the flowering period of *P. mume* and discern the location and expression of glycoside synthesis genes, we extracted and enzymatically hydrolyzed GBVs of *P. mume* and then utilized gas chromatography–mass spectrometry (GC–MS) to characterize and analyze the types and contents of GBV glycosides. Further, we identified and classified the members of the UGT gene family of *P. mume* using the bioinformatic method and analyzed the correlation between the expression of the UGT family genes in *P. mume* and the changes in glycosidic content. The results showed that the benzenoids were the main aromatic substance that was glycosylated during flowering in *P. mume* and that glycosidic benzaldehyde was the most prevalent compound in different flower parts and at different flowering stages. The titer of glycoside benzaldehyde gradually increased during the bud stage and reached the highest level at the big bud stage (999.6 μg·g^−1^). Significantly, titers of glycoside benzaldehyde significantly decreased and stabilized after flowering while the level of free benzaldehyde, in contrast, significantly increased and then reached a plateau after the flowering process was completed. A total of 155 UGT family genes were identified in the *P. mume* genome, which were divided into 13 subfamilies (A–E, G–N); according to the classification of *Arabidopsis thaliana* UGT gene subfamilies, the L subfamily contains 17 genes. The transcriptome analysis showed that *PmUGTL9* and *PmUGTL13* were highly expressed in the bud stage and were strongly correlated with the content of the glycosidic form of benzaldehyde at all stages of flowering. This study provides a theoretical basis to elucidate the function of UGT family genes in *P. mume* during flower development, to explore the mechanism of the storage and transportation of aromatic compounds in flower tissues, and to exploit industrial applications of aromatic products from *P. mume*.

## 1. Introduction

Plants produce terpenoids, phenols, alkaloids, etc., during metabolism [1,2], and these secondary metabolites play important roles in normal plant growth and metabolism and in response to biotic and abiotic stresses [3,4]. The large intracellular accumulation of secondary metabolites disrupts the cell membrane structure and causes toxic effects on the plant cells [5]. An effective way for plants to cope with excessive intracellular secondary metabolites is to modify them into non-toxic and low-active glycoside-bound compounds, a process that requires the transfer of sugar groups into secondary metabolites with the aid of glycosyltransferases [6,7]. Among them, uridine diphosphate glycosyltransferase (UGT) can catalyze the glycosylation of plant secondary metabolites using uridine diphosphate sugar molecules as donors [8]. A total of 14 UGT subfamilies were identified in *Arabidopsis thaliana*. A benzoate substrate screening of 90 UGT recombinant proteins in *Arabidopsis thaliana* by Alexandra et al. revealed that members of the L subfamily are highly specific for O-glucoside formation, suggesting that the uridine diphosphate glycosyltransferase L-subfamily (UGTL) recognizes phenylpropane substrates and modifies them to form glycosidic bonds [9].

Small volatile compounds released within plant flower tissues exert complex effects including the exchange of information between organisms, the attraction of beneficial insects, and for biodefenses [10,11]. Aromatic volatiles exist in three forms: the volatile state released into the air, the free state located in cellular endogenous spaces, and the glycoside-bound states (GBVs) that are formed by linking with sugar groups [12]. With the discovery of monoterpene glycoside compounds in roses, several aromatic glycoside compounds have been isolated and identified in grapes and tea, with most GBV glycoside structures being β-D-glucoside and α-L-rhamnoside [6,13,14,15]. Changes in tea, wine, fruit, and floral aromas in production processes are mostly related to the transformation of GBV [16,17,18,19]. Glycosidic phenylpropane volatiles during tomato growth and development were highest at fruit ripening, and tomato fruit flavor quality was enhanced when the relevant β-glucosidase gene was overexpressed, suggesting that aromatic volatiles can be stored in tomatoes in the form of GBV [20].

*Prunus mume* is one of China’s ten most renowned flowers, with rich cultural connections and widespread popularity [21]. Benzenoid substances are secondary metabolites of phenylalanine generated by several modifications catalyzed by phenylalanine ammonium lyase (PAL) [22], in which benzaldehyde can be reduced to benzyl alcohol, which can also be catalyzed by acetyltransferase to generate benzyl acetate. Several components of this pathway are volatile and collectively constitute the characteristic aroma of *P. mume* [23].

Benzaldehyde is produced in large quantities at sites of mechanical damage in plants [24]. This compound inhibits the germination of fungal spores [25] and is a major component of new agricultural insecticides [26]. All these studies suggest that benzaldehyde plays an important role in plant defense. Hao [23] identified and quantitatively analyzed the volatile and free aromatic constituents in *P. mume* and found that the free-state content of the main aromatic constituents of *P. mume* was significantly greater than the volatile amount. The free-form aromatic components exhibit the highest benzaldehyde content, yet their volatilization efficiency is remarkably low [27], suggesting that, in *P. mume*, the predominant form of benzaldehyde is in its free state. Mechanisms exist for the interconversion of free and glycosidic states of defense compounds in plant cells [28], for example, when *Camellia sinensis* is infected with anthracnose, geraniol and benzyl alcohol in the form of glycosides are hydrolyzed and released by endogenous glycosidases to achieve an antimicrobial effect [29]. We speculate that there may also be an interconversion between the glycosidic and free states of benzaldehyde in *P. mume* as a way to maintain benzaldehyde at a certain concentration to achieve the defense function.

The mechanism of interconversion between free and glycosidic floral components in *P. mume* is not well understood. In this study, we identified and analyzed the glycosidic aromatic substances in different anthesis stages and parts using GC–MS, which was combined with the content of free aromatic substances to summarize the change rule of glycosidic benzaldehyde in different anthesis stages. UGT family members were identified from *P. mume*, and a phylogenetic tree was constructed to allow for the analysis of chromosomal localization and collinearity using bioinformatics. This study further utilized transcriptome data to screen the *UGTL* genes specifically expressed at different times of *P. mume*, which provided a reference for elucidating the mechanism of *UGTL* genes involved in the synthesis of aromatic substances’ glycosidic state in *P. mume.*

## 2. Results

### 2.1. Glycosidic Aromatic Substances of P. mume in Different Anthesis Stages

**Figure 1:**
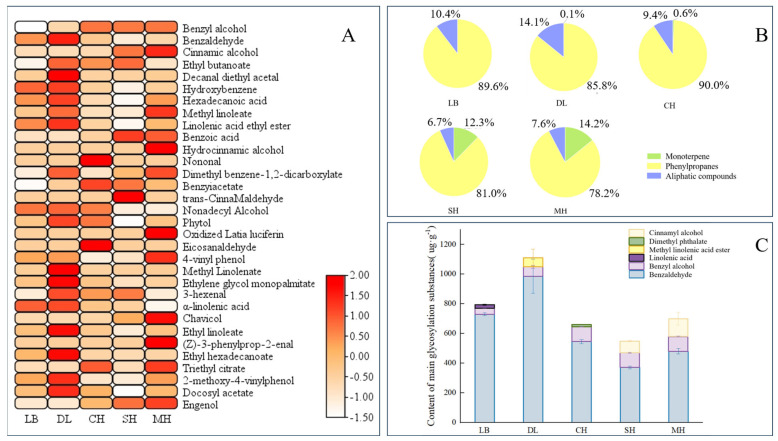
Analysis of aroma glycosides at different blooming stages of *P. mume*. (**A**): Heatmap analysis of aroma glycosides at different blooming stages of *P. mume*. Each column represents a flowering period. The emission values were normalized using the log_10_ transformation. The color of the heatmap ranges from white (value, −1.5) to red (value, 2) in the natural logarithmic scale. Data are presented with means of three biological replicates. (**B**): Relative content of aromatic glycosidic properties at different blooming stages of *P. mume.* (**C**): Glucoside content of main aromatic substances at different blooming stages of *P. mume.* The top three glycosidic substances at each flowering stage are listed in the figure. LB, dew petal stage; DL, large bud stage; CH, first bloom stage; SH, full bloom stage; MH, last bloom stage.

Glycosidic substances were extracted and analyzed from *P. mume* of different anthesis stages, and a total of 27 glycosidic aromatic substances were detected. According to the glycosidic compound classification method, the glycosidic aromatic substances in *P. mume* were classified into three categories, namely, monoterpenes, phenylpropanoids, and aliphatic compounds [30]. These included 4 monoterpenes, 10 phenylpropanes, and 13 aliphatic compounds. A total of 20 substances were glycosylated at the big bud stage, and their contents were relatively higher compared to other anthesis stages. The largest number of aromatic substances was glycosylated at the primordial stage, with 25 substances glycosylated, including 3 monoterpenes, 9 phenylpropanes, and 13 aliphatic compounds (Figure 1A).

The relative contents of glycosylated phenylpropanes were the highest at different anthesis stages of *P. mume*, and all of them were greater than 78%, with the highest relative content of 89.6% in the early flowering period, which is when the main glycosylated aromatic substances were found in *P. mume*. Glycosidic monoterpenes started appearing at the large bud stage and gradually increased with the flowering process, with the highest content of 14.2% compared to other flowering stages at the last bloom stage (Figure 1B). Benzaldehyde and benzyl alcohol were the main glycosidic aromatic substances in *P. mume*, and the content of glycosidic benzaldehyde was the highest in different anthesis stages, with the highest content in the big bud stage showing a trend of gradual decrease with the continuation of the anthesis stage (Figure 1C). By comparing the types and absolute and relative contents of glycosidic states in different anthesis stages, the results are consistent with the fact that benzene ring-like substances are the predominant volatile aroma components in developing flower tissues of *P. mume*, indicating that *P. mume* could store benzyl alcohol and benzaldehyde in the form of glycosidic states in the flora.

### 2.2. Quantitative Analysis of Endogenous Benzaldehyde in Different Floral Parts of P. mume

**Figure 2:**
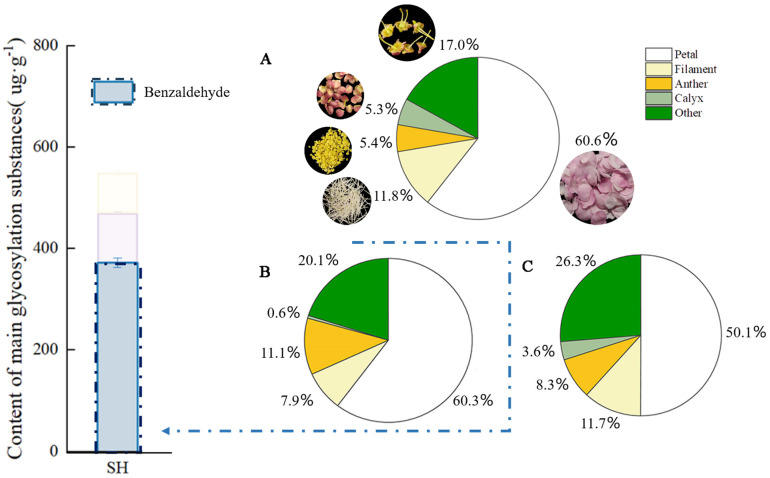
Free and glycoside benzaldehyde quantitative analysis in different parts of *P. mume.* The blue bars in the histogram represent the glycosidic state benzaldehyde content in the full bloom stage of *P. mume* (**A**): Proportion of weight of different parts of flowers. The circular picture around the pie chart shows the materials used. (**B**): The content of glycoside benzaldehyde in different parts of flowers. (**C**): The content of free benzaldehyde in different parts of flowers. SH, full bloom stage.

Quantitative analyses were conducted on the mass, glycosidic, and free benzaldehyde contents in the five organs of *P. mume* at full bloom. As can be seen from Figure 2, petals had the largest mass proportion of 60.6% among the parts of *P. mume* at full bloom, while their glycosidic and free benzaldehyde contents were the highest, at 60.3% and 50.1%, respectively, followed by benzaldehyde endogenous contents in other flower parts (disk + pistil). The mass ratio of the anther part was 5.4%, but its content of glycosidic and free benzaldehyde reached 11.1% and 8.3%, respectively; the mass ratio of the calyx was similar to that of the anther, but its content of glycosidic and free benzaldehyde was only 0.6% and 3.6%, respectively; 11.3% of free benzaldehyde and 7.9% of glycosidic benzaldehyde were detected in the filament.

### 2.3. Quantitative Analysis of Glycosidic State of Key Components of P. mume in Different Anthesis Stages

**Figure 3:**
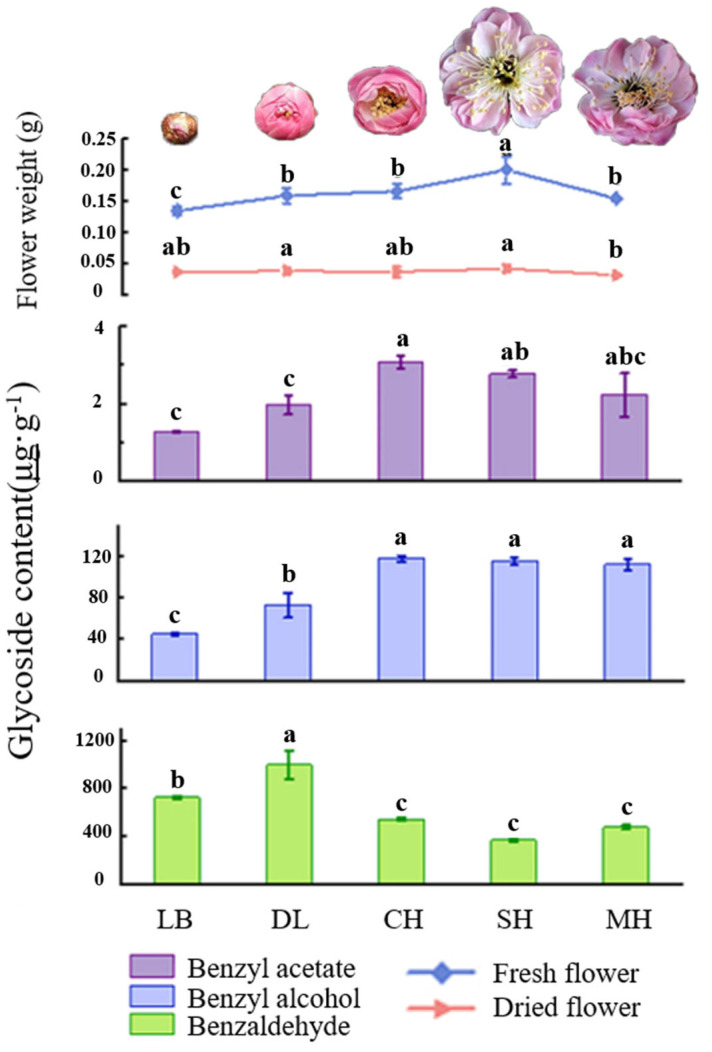
Quality and key glycosylated aromatic components’ quantitative analysis at different blooming stages of *P. mume.* Data are mean ± standard deviation (SD) calculated from three biological replicates. Vertical lines represent standard deviation. The different lowercase letters indicate significant difference at 0.05 level. LB, dew petal stage; DL, large bud stage; CH, first bloom stage; SH, full bloom stage; MH, last bloom stage. The *P. mume* in the picture is a display of material from each stage of flowering.

Phenylpropanes were the main aromatic substances in floral tissues of *P. mume*, as was found after analyzing the glycosidic state substances of the characteristic aroma after hydrolysis by β-glucosidase. The content of glycosidic benzaldehyde was significantly higher than that of benzyl alcohol and benzyl acetate in flower tissues at different developmental stages of *P. mume*, in which the content of glycosidic benzaldehyde showed a general trend of increasing and then decreasing, and the content of benzaldehyde in the dew petal stage and bud stage was significantly higher than that in other stages and reached the maximum value of 999.6 μg·g^−1^ in the big bud period. The glycosidic contents of benzyl alcohol and benzyl acetate increased gradually over the period, and they were significantly higher than those in the first-flowering stage and the last-flowering stage, reaching a maximum of 118.37 μg·g^−1^ and 3.1 μg·g^−1^, respectively, in the first-flowering stage. The differences between the first-flowering stage and the last-flowering stage were not significant. The fresh weight of the flowers gradually increased with the flowering stage and reached their maximum at the full bloom stage. This was consistent with the trend of the contents of benzyl alcohol and benzyl acetate in the glycosidic states, and the fresh and dry weights of the flowers at the final flowering stage were both significantly lower than those at the full bloom stage (Figure 3).

### 2.4. Quantitative Analysis of Endogenous Benzaldehyde at Different Anthesis Stages of P. mume

**Figure 4:**
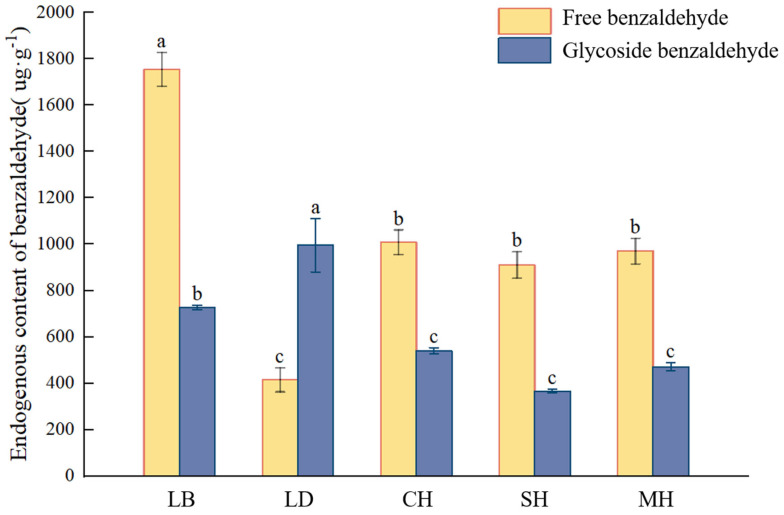
Volatile, free, and glycosylated benzaldehyde quantitative analyses at different blooming stages of *P. mume.* Data are mean ± standard deviation (SD) calculated from three biological replicates. Vertical lines represent standard deviation. The different lowercase letters indicate significant difference at 0.05 level. LB, dew petal stage; DL, large bud stage; CH, first bloom stage; SH, full bloom stage; MH, last bloom stage.

This study quantified the content of glycosidic benzaldehyde at five flowering anthesis stages of *P. mume* and analyzed it in combination with the free state and volatile amount of benzaldehyde in each period. As can be seen from Figure 4, the free benzaldehyde content was significantly higher in the dew petal period than in other anthesis stages, reaching 1754.16 μg·g^−1^. As the flower buds expanded to large buds, the free-state content decreased abruptly, and the content of glycosidic benzaldehyde in the same period was the highest compared to that in the other anthesis stages, reaching 995.48 μg·g^−1^. The free benzaldehyde content stabilized after the blooming period, while the glycosidic benzaldehyde content continued to show a decreasing trend as the flowering period progressed.

### 2.5. Identification and Subfamily Classification of PmUGT Family Members

**Figure 5:**
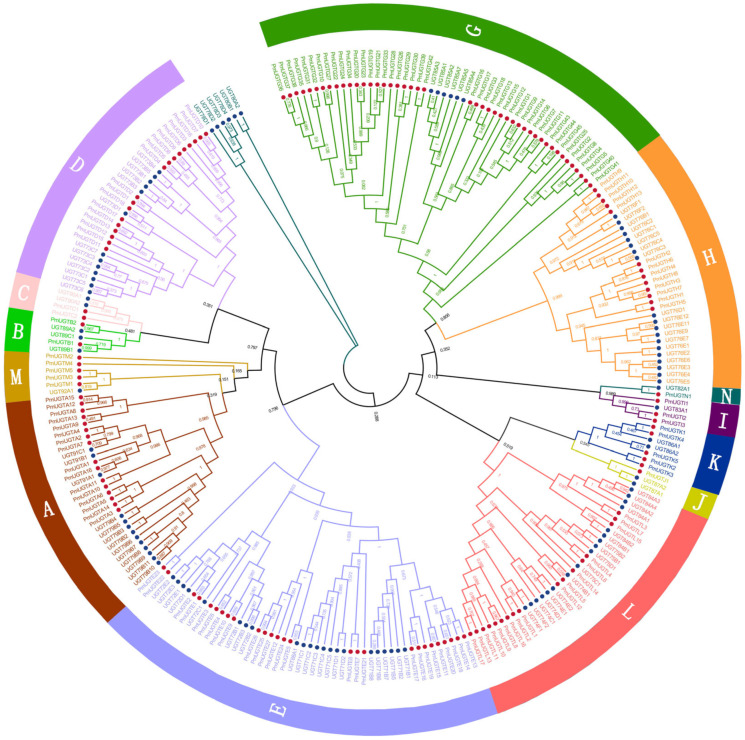
Phylogenetic tree of *PmUGTs* and *AtUGTs*. Phylogenetic trees were constructed using the neighbor-joining (NJ) method and repeated 1000 times. All *PmUGTs* were classified into 13 subfamilies (A–E, G–N), and each subfamily is indicated by a different color.

In order to comprehensively characterize the UGT gene family in *P. mume*, the Pfam structural model of UGT proteins and 114 UGT protein sequences in *Arabidopsis thaliana* were used as references for comparison and screening, and 155 members of the PmUGTs family were finally obtained. A phylogenetic tree of the UGT family in *P. mume* and *Arabidopsis* was constructed using the NJ method of MEGA11, and the results show that the UGT family genes were classified into 13 subfamilies (Figure 5), including *UGTA*-*E* and *UGTG-N*. The *PmUGTG* subfamily had the largest number of members, with 45 members, which accounted for 29.03% of the whole UGT family, and the *PmUGTJ* subfamily had the smallest number of members, with only 1 member. The *PmUGTE* and *PmUGTL* subfamilies had 27 and 17 members, respectively. No member of subfamily F was identified in *P. mume*.

### 2.6. Chromosome Localization and Colinearity Analysis of the PmUGT Family

**Figure 6:**
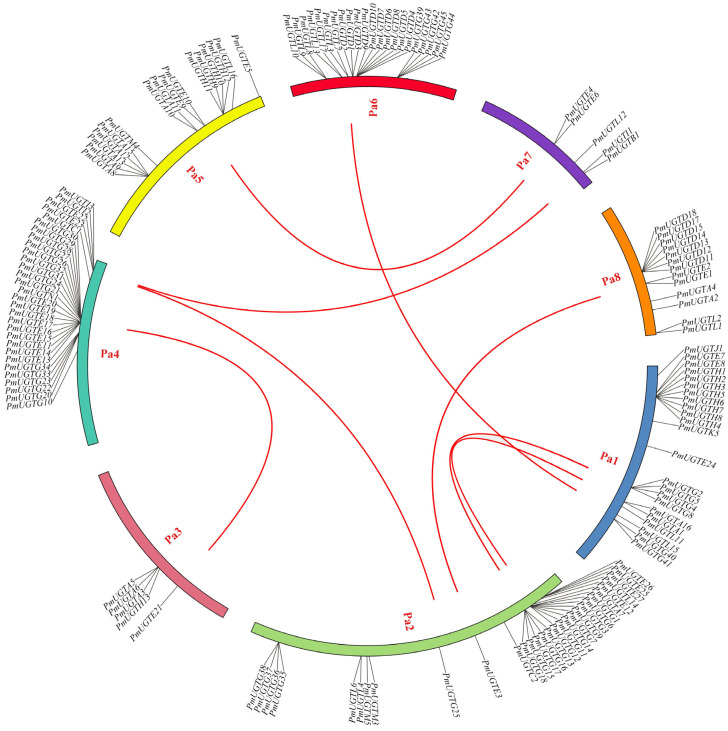
The chromosome distribution and collinearity analysis of *PmUGT* family genes. Individual chromosomes are represented by different colors, and the red lines in the figure represent segmental repeats.

According to the chromosome localization analysis of *PmUGTs* (Figure 6), 144 of the 155 *PmUGTs* genes were distributed on eight chromosomes, and the number of genes localized on each chromosome was uneven, with the largest number of *PmUGTs* members on chromosomes 4 and 2, both with 30 members, and only 5 *PmUGTs* members on chromosomes 3 and 7. The distribution of different subfamily members on the chromosomes was irregular, with the *PmUGTL* members distributed on all six chromosomes, but with a maximum of seven *PmUGTs* members on chromosome 6. Some family members existed as gene clusters, such as *PmUGTR13-20* on chromosome 4 with a total of eight E family members in one cluster and *PmUGTH1-8* on chromosome 1 in one cluster, while *PmUGTG*′ family members existed in gene clusters on chromosomes 2, 4, and 6.

### 2.7. Expression Analysis of PmUGTL Genes at Different Anthesis Stages of P. mume

**Figure 7:**
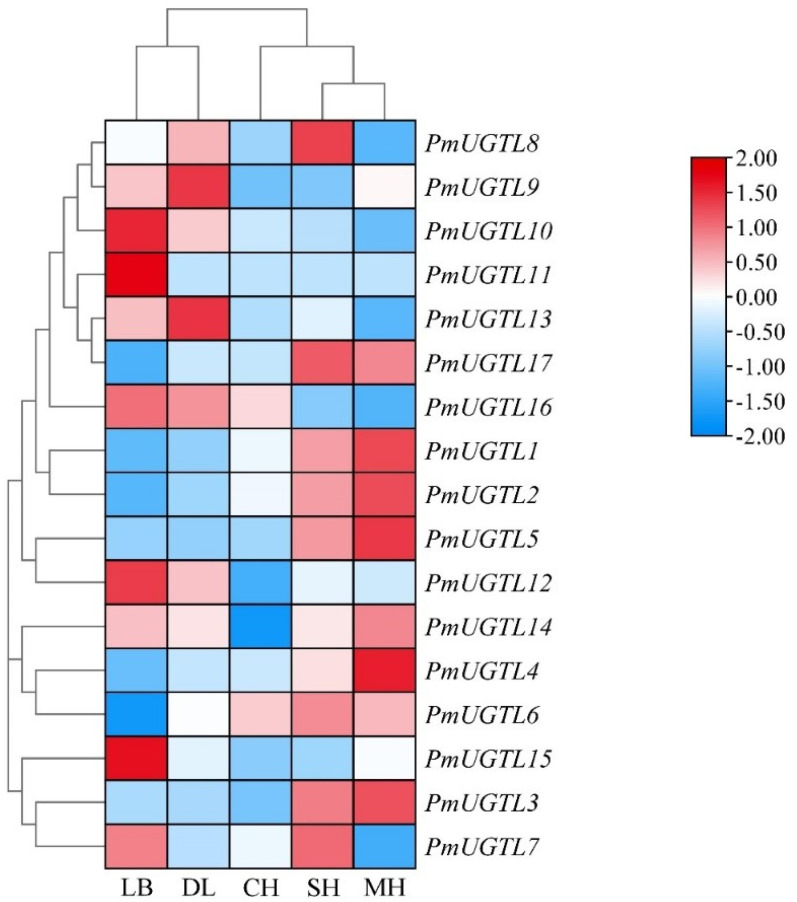
Expression calorimetry of *PmUGTLs* gene of *P. mume* at different stages. The emission values were normalized through log_10_ transformation. The color of the heatmap ranges from blue (value, −2) to red (value, 2) in the natural logarithmic scale. Data are presented as means of three biological replicates. LB, dew petal stage; DL, large bud stage; CH, first bloom stage; SH, full bloom stage; MH, last bloom stage.

In order to further study the *UGTL* genes’ expression patterns at different anthesis stages of *P. mume*, 17 *UGTL* genes at five anthesis stages were visualized based on transcriptome sequencing data (Figure 7). The results show that the expressions of *PmUGTL9* and *PmUGTL13* were higher at the large bud stage than at other anthesis stages; that the expressions of *PmUGTL10*, *11*, *12*, and *15* were higher at the dewlap stage; and that the expression of *PmUGTL16* was the highest at the primordial stage compared with other genes. *PmUGTL1*, *2*, *3*, *4*, and *5* were all expressed at higher levels at the last bloom stage.

### 2.8. Correlation Analysis between Benzaldehyde Glycosidation and PmUGTL Gene Expression in Prunus mume

**Figure 8:**
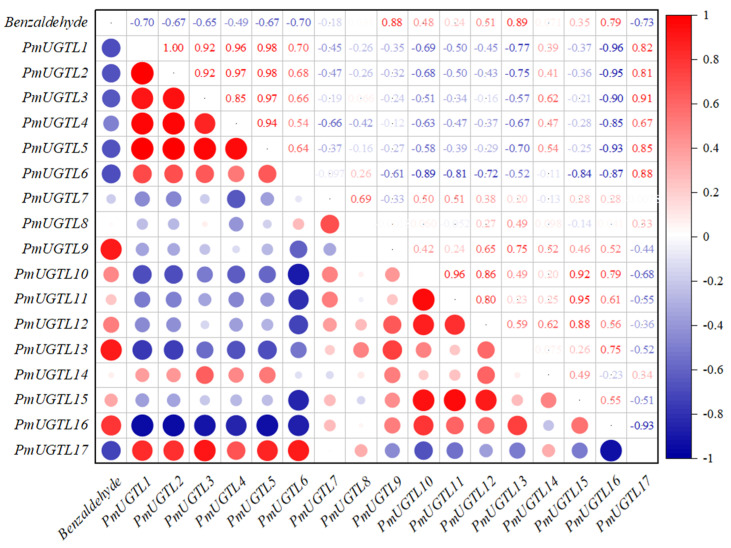
Correlation analysis between synthesis efficiency of key glycosides and PmUGTL gene expression in *P. mume*. The emission values were normalized through log_10_ transformation. The color of the heatmap ranges from dark blue (value, −1) to red (value, 1) in the natural logarithmic scale. Data are presented as means of three biological replicates.

A correlation analysis between the glycosidic benzaldehyde syntheses at each period of *P. mume* and *PmUGTL* genes’ expression was conducted (Figure 8). The glycosidic benzaldehyde synthesis was strongly positively correlated with *PmUGTL9* and *13* positively correlated with *PmUGTL16*. Notably, *PmUGTL1*, *2*, *3*, *5*, and *6* were all strongly negatively correlated with glycosidic benzaldehyde synthesis, and the expression levels of *PmUGTL1*, *2*, and *5* were all relatively high at the last flowering stage.

### 2.9. Validation of qRT-RCR of Key Genes of PmUGTLs

**Figure 9:**
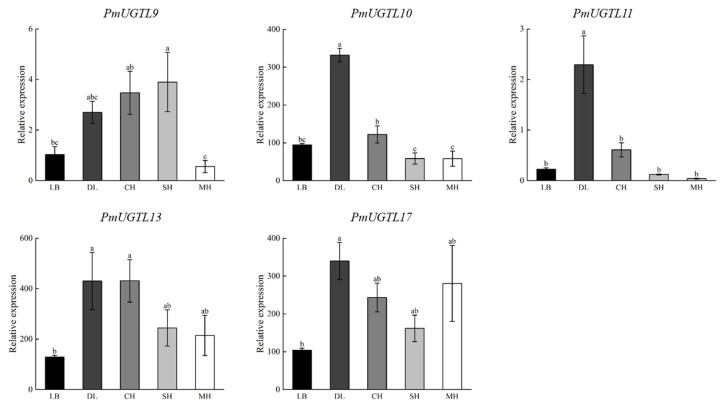
qRT-RCR of key *P. mume* gene. Data are mean ± standard deviation (SD) calculated from three biological replicates. Vertical lines represent standard deviation. The different lowercase letters indicate significant diference at 0.05 level. LB, dew petal stage; DL, large bud stage; CH, first bloom stage; SH, full bloom stage; MH, last bloom stage.

Combining the results of the correlation analysis, gene expression calorimetry, and changes in the period of benzaldehyde glycosylation, *PmUGTL9* and *13* were identified as candidate genes, and qRT-PCR was performed for the candidate genes and their homologous genes (Figure 9). It was found that the expression levels of *PmUGTL10*, *13*, and *17* were significantly higher than that of other genes, as *PmUGTL10* and *13* showed the same trend with benzaldehyde glycosidation, and *PmUGTL17* was highly expressed in the end-flowering period compared to other genes.

## 3. Discussion

Plants emit an overabundance of volatile compounds that play different roles in plant protection when the free aromatics accumulate in the cells at a certain concentration [31,32]. Plants can also convert excess volatiles, which are toxic to cells, into GBVs as a form of protection when plants suffer abiotic or biotic stress [33]. A previous study found that benzaldehyde is prevalent in plants of the Rosaceae family and that most of them defend themselves by accumulating benzaldehyde [34]. Large amounts of amygdalin are present in peach kernels and almonds. Once the cell structure is broken down, the amygdalin in the vesicles is hydrolyzed by glycoside-degrading enzymes in the cytoplasm, producing bitter-tasting benzaldehyde and toxic prussic acid [28]. The results of this study indicate that benzaldehyde is the main glycosidic substance present in *P. mume*. The glycosidic and free benzaldehyde contents constituted 60.3% and 50.1% of the total amount of benzaldehyde in full-bloom petals, respectively. In comparison, the concentrations of both glycosidic and free benzaldehyde were significantly higher in the anthers than in other parts of *P. mume*.

The site where the synthesis of GBV for partial defense takes place is not the same as that for storage. Defensive cyanogenic glycosides can be transported to the roots via the phloem in cassava leaves [35]. Brown found that newborn leaves and ungerminated seeds of *Arabidopsis* had the highest concentrations of mustard oleoresin, whereas germinated seeds and senescent leaves had significantly reduced levels of mustard oil glycosides [36]. Doyle suggests that plants move defenses to the most protective tissues at different times [37]. The glycosylation of defense substances coordinates in vivo homeostasis and in vitro defense functions in plants, and defense through the hydrolysis of toxic substances is a convenient strategy for plants to cope with stress [38]. In *P. mume*, the anthers constitute a very small percentage by weight, but the anthers contain higher concentrations of benzaldehyde in the free and glycosidic forms. A previous study found that benzaldehyde was the component with the highest relative volatilization in the anthers of *P. mume*, with a volatilization of 65.20%, which was significantly higher than the remaining four parts of *P. mume* [27]. Therefore, we hypothesize that *P. mume* attracts insects by releasing aromatic substances and stores high concentrations of free and glycosidic benzaldehyde in the anthers to protect the anthers, which is effective in preventing insects from eating the anthers while pollinating them through insects.

Based on our findings, we hypothesize that *P. mume* stores large amounts of glycosidic and free benzaldehyde in the anthers during flowering to prevent insects from nibbling the pollen. In the present study, glycosidic and free benzaldehyde in *P. mume* were found to show different patterns of change over time. The free benzaldehyde content continued to decrease, while the glycosidic benzaldehyde content continued to increase and was significantly higher than at other times, starting in bud development and continuing to the large-bud stage. This suggests that there is significant benzaldehyde glycosylation during the budding phase, which is consistent with the period when GBV production is high in the rose [13]. The content of glycosidic benzaldehyde significantly decreased and showed a stable level after flowering, while the content of free benzaldehyde significantly increased and showed a stable level after flowering, which is consistent with the pattern of change of benzene GBV obtained by John et al. in aromatic tobacco [39]. During the flowering period, *P. mume* is able to volatilize a large number of aromatic compounds [40]. Benzaldehyde is the upstream substrate of benzyl alcohol and phenylmethyl acetate, which are the main aromatic components in the synthesis of *P. mume* [23]. Based on the above, we hypothesize that a large amount of glycosidic benzaldehyde can act as a reserve of aromatic precursors during the bud stage and that the glycosidic state is gradually hydrolyzed during flowering to replenish the free benzaldehyde for phenylmethyl acetate synthesis.

Widespread in plants, UGT is involved in regulating metabolites such as flavonoids, terpenoids, phenylpropanoids, and phytohormones with diverse biological functions [41]. The UGT family has been identified in several species. A total of 99 were identified in *Arabidopsis thaliana* [42], and 168 in peach, which is more closely related to *P. mume*. [43]. In our study, however, 155 UGT family genes were identified in *P. mume*. The *P. mume UGTs* were classified into subfamilies with reference to *Arabidopsis thaliana*. In *Arabidopsis*, the L subfamily recognizes phenylpropane as a substrate. A large amount of free benzaldehyde, which is structurally similar to phenylpropane compounds, was found in *P. mume*, we chose to target the *UGTL* subfamily in *P. mume* for further analysis. Correlating the gene expression of *PmUGTLs* with the content of glycosidic benzaldehyde in this study, we obtained *PmUGTL9* and *PmUGTL13*, which had a very strong positive correlation between gene expression and synthesis efficiency, and *PmUGTL1*, *2*, *3*, *5*, and *6*, which all had strong negative correlations with the amount of glycosidic benzaldehyde synthesized, and all five genes were highly expressed at the time of the end of flowering, which is not consistent with the time of the massive glycosidation of benzaldehyde. *UGT85A19* (Accession: B2XBQ5) in *Prunus dulcis* is homologous to candidate gene *PmUGTL13*, which is involved in the synthesis of the (R)-bitter amygdalin precursor and associated with the strong bitter flavor of almonds [44]. Benzaldehyde is a substrate for the synthesis of bittersweet amygdalin and has a characteristic bitter amygdaloid flavor. Therefore, the gene *PmUGTL13* is more likely to be associated with the synthesis of the glycosidic state of benzaldehyde in *P. mume*.

## 4. Materials and Methods

### 4.1. Plant Materials

Multiple healthy and vigorous ten-year-old *P. mume* on the campus of Shanxi Agricultural University were used. During the flowering period from February to March 2022, the flowers were collected from 7 to 9 a.m. on a sunny morning at the dew petal stage (LB), large bud stage (DL), first bloom stage (CH), full bloom stage (SH), and last bloom stage (MH) (Figure 3). The full-bloom-stage flowers that were of uniform sizes were selected and rapidly divided into petals, filaments, anthers, calyx, and other parts on ice (Figure 2), and they were quickly frozen in liquid nitrogen and stored in an ultra-low temperature refrigerator at −80 °C.

### 4.2. Instruments and Reagents

β-D-glucosidase was purchased from Shanghai Yuanye Biotechnology Co., Ltd., Shanghai, China; methanol (chromatographic pure), ethyl acetate (chromatographic pure), citric acid, disodium hydrogen phosphate, and anhydrous sodium carbonate were obtained from Tianjin Damao Chemical Reagent Factory, Tianjin, China; concentrator plus vacuum concentrator, ultra-low temperature refrigerator, electronic balance, ultrasonic cleaner, pipette gun were obtained from Eppendorf, Hamburg, Germany; thermostatic bath was obtained from PolyScience, Niles, IL, USA; acid meter, Trace1300 gas chromatograph, Trace ISQ mass spectrometer (GC-MS), and NanoDrop 2000 nucleic acid analyzer were obtained from Thermo, Waltham, MA, USA; high-efficiency plant total RNA extraction kit, UnionScript First-strand cDNA synthesis mix for qPCR kit, and real-time fluorescence quantitative SYBR GreenⅠMaster kit were purchased from Beijing Jinsha Biological Co., Ltd., Beijing, China; LightCycler 480 quantitative PCR instrument was obtained from Roche, Basel, Switzerland.

### 4.3. Extraction of Glycosidic Aromatic Substances

The cinnamon glycoside extraction method was referred to and optimized, we inactivated the endogenous enzymes of *P. mume* to eliminate the effect of endogenous enzymes on the results [17]. The material was weighed as 0.5 g samples from the different anthesis stages, with 0.2 g samples of the different parts of *P. mume*, which were fully ground together with liquid nitrogen into a glass test tube. To this, 5 mL of 80% methanol solution was added, and heated with an alcohol lamp for 2 min, all of which was transferred to a 10 mL centrifuge tube for ultrasonic shaking for 15 min to assist the extraction, followed by a low-temperature centrifugation of 10,000× *g* rpm at 4 °C for 15 min. Then, the supernatant was partitioned into 2 mL centrifuge tubes and placed in a vacuum concentrator and then concentrated in a vacuum at 30 °C until it was completely dry to remove the methanol.

### 4.4. Hydrolysis of Glycosidic Aromatic Substances

The crude extracted glycosides were dissolved with 2 mL of 0.2 mol·L^−1^ citric acid and 0.2 mol·L^−1^ disodium hydrogen phosphate buffer, to which 5 U β-glucosidase was added. This was covered with 3 mL of ethyl acetate and fully extracted by using ethyl acetate in a 37 °C water bath for 24 h. The extracts were dried with anhydrous sodium carbonate, filtered with an organic system filtration membrane, and transferred to the liquid feed vials for GC–MS analysis.

### 4.5. GC-MS Analysis

The analysis of the endogenous components was performed using GC–MS, and the GC conditions were as follows: the carrier gas was helium, and the VF-5MS column (30 m × 0.25 mm × 0.25 μm) was used as the chromatographic column. The heating-up program was set as follows: the starting temperature was 40 °C for 2 min, the temperature was raised to 180 °C at the rate of 4 °C·min^−1^ and maintained for 3 min, and then the temperature was raised to 220 °C at a rate of 10 °C min^−1^. The MS conditions were as follows: the ionization mode was EI, 45–500 amu for the scan range for the mass-to-charge ratio (*m*/*z*), 70 eV for the electronic energy, and the GC–MS interface temperature and the ion source temperature were both 250 °C. The endogenous components in *P. mume* were identified using the X caliber software system(v2.0.7); the components were determined by searching through the NIST08 standard spectral library and with manual spectral analysis. The aromatic components were quantified using the external standard method.

### 4.6. Statistics and Analysis of Data

Three replications were performed for each sample, and the experimental data were integrated and calculated using Microsoft Excel 2019 and analyzed for significance, while Duncan’s multiple comparisons (*p* < 0.05) were used using SPSS 22.0 software and plotted using Origin Pro 2021.

### 4.7. Transcriptome Measurement of P. mume at Different Nthesis Stages

Total RNA was extracted from five flowering stages of *P. mume*, and the samples were set up in three sets of biological replicates for transcriptome sequencing using the Illumina HiSeq^TM^2500 platform (Bemac Biotech Co., Ltd., Imabari, Japan). The sequencing results were analyzed using BMK Cloud (www.biocloud.net (accessed on 1 November 2022)). The sequencing results were uploaded to the SRA database of NCBI, GenBank: PRJNA905928.

### 4.8. Identification of UGT Gene Family Members

*P. mume* whole genome data were obtained from the *P. mume* genome database (http://Prunusmumegenome.bjfu.edu.cn (accessed on 4 March 2023)), and UGT protein structural domain files (PF00201,UDPGT.HMM) were obtained from the Pfam protein structure database (http://pfam.xfam.org/ (accessed on 4 March 2023)) [45]. HMMER 3.0 was used to construct the UGT Hidden Markov Model and was used in the *P. mume* Genome Protein Database for comparative searches with an E-value of 10^−5^. Arabidopsis UGT transporter protein sequences were obtained from the Arabidopsis thaliana database (https://www.arabidopsis.org/ (accessed on 5 March 2023)), and the *P. mume* Genome Protein Database was searched with BLASTP. The resultant files were submitted to the NCBI Batch CD-search (https://www.ncbi.nlm.nih.gov/ (accessed on 8 March 2023)) database, where candidate sequences were screened for conserved structural domains, and sequences with incomplete PSPG box structures were removed.

### 4.9. Multiple Sequence Alignment and Subfamily Classification of the UGT Gene Family

The identified *PmUGT* gene candidate sequences were compared with the protein sequences of *AtUGT* family members using ClustalW, and the comparison results were used to construct a phylogenetic tree of the *PmUGT* gene family using the neighbor-joining method (NJ) in the MEGA11 software, with the bootstrap value set to 1000 times. The Poisson model was selected for the alternative model, partial deletion was used, and the default settings were used for the rest of the parameters. Referring to the distribution of Arabidopsis subfamily UGT genes on the phylogenetic tree, *PmUGTs* were categorized into 13 subfamilies (*PmUGTA-E, PmUGTG-N*).

### 4.10. Chromosomal Localization and Colinearity Analysis of the UGT Gene

All *P. mume* UGT family genes were localized in chromosomes using Circos based on *P. mume* genome annotation information. Sequence pairs were made within the *P. mume* genome using the TBtools sequence comparison tool Blast Compare 2 Seq (E-value < 10^−5^) to obtain homologous gene pairs, which were analyzed using the covariance analysis tool MCScanX to obtain covariance regions within the genome, and the intraspecies relationships of the UGT family genes were visualized using TBtools(v2.003).

### 4.11. Analysis of the Expression of UGTLs Genes in P. mume at Different Anthesis Stages with Time

Gene expression analysis was carried out using transcriptome data from five anthesis stages of *P. mume*. Based on the data of differentially expressed genes, the RPKM values of gene expression of *PmUGTLs* were extracted, and the heatmap of gene expression was drawn. A correlation analysis of the transcriptome data of *P. mume* at different anthesis stages with the benzaldehyde glycosidation content at different anthesis stages was performed and plotted with Origin Pro 2021.

### 4.12. Quantitative Expression Analysis of Candidate Genes for UGTLs

The software Primer Premier 5 was used to design the qRT-PCR primers for the key genes, and the specificity of the primers was verified using the BLAST online tool on the NCBI website. The high-efficiency plant total RNA extraction kit was selected to extract the total RNA of Prunus mume at each flowering stage, the quality of the resulting RNA was examined using NanoDrop 2000 Nucleic Acid Meter, repeated three times for each group. The unionscript first-strand cDNA synthesis mix for the qPCR kit was used for the first-strand reverse transcription, and the qRT-PCR of the key genes was performed with the real-time fluorescence quantitative SYBR GreenⅠMaster kit. The quantification was carried out by the Roche Light Cycler 480 instrument, and the UBC gene was used as an internal reference gene [46]. The reaction procedures were as follows: pre-denaturation at 95 °C for 3 min; denaturation at 94 °C for 20 s; 60 °C annealing and extension for 34 s; a total of 40 cycles. The gene relative expression level was calculated using the 2^−ΔΔCt^ method with the *P. mume* dew petal stage as the control [47].

## 5. Conclusions

*P. mume* is a unique woody aromatic plant, and phenylpropanoids/benzenoids are the main components of the GBVs. The content of glycoside benzaldehyde was found to be the highest at different stages and parts of the plant, and the titer of glycoside benzaldehyde gradually increased during the bud stage and reached the highest level at the big bud stage. We identified 155 UGT family members in *P. mume* and divided these family members into 13 subfamilies. Furthermore, combined with the correlation analysis between the synthesis efficiency of glycosides and the expression of *PmUGTL* genes in the different blooming stages, our results indicate that *PmUGTL9* and *13* were the key candidate genes. The expression profile of *PmUGTL13* was extremely consistent with the synthesis efficiency of benzaldehyde glycosides, so we posit that *PmUGTL13* is largely involved in the synthesis of benzaldehyde glycosides. This research further elucidates the important role played by benzaldehyde glycosidation in maintaining stable intracellular benzaldehyde concentrations, floral defense, and the stable volatilization of aromatic components. In addition, it provides a theoretical basis to elucidate the function of UGT family genes in *P. mume* during flower development, to explore the mechanism of the storage and transportation of aromatic compounds in flower tissues, and to exploit industrial applications of aromatic products from *P. mume*.

## Data Availability

Data reported are available in Section 2 and Supplementary Materials.

## Supplementary Materials

The following supporting information can be downloaded at: https://www.mdpi.com/article/10.3390/plants13050703/s1, Table S1: Gene primer sequence, Table S2: Absolute content of glycosidic aromatic substances at different anthesis stages of *P. mume*, Table S3: Absolute content of glycosidic aromatic substances in different parts of *P. mume,* Table S4: Physicochemical properties and prediction of subcellular location in *P. mume.*
